# Subthreshold amyloid deposition, cerebral small vessel disease, and functional brain network disruption in delayed cognitive decline after stroke

**DOI:** 10.3389/fnagi.2024.1430408

**Published:** 2024-09-16

**Authors:** Jae-Sung Lim, Jae-Joong Lee, Geon Ha Kim, Hang-Rai Kim, Dong Woo Shin, Keon-Joo Lee, Min Jae Baek, Eunvin Ko, Beom Joon Kim, SangYun Kim, Wi-Sun Ryu, Jinyong Chung, Dong-Eog Kim, Philip B. Gorelick, Choong-Wan Woo, Hee-Joon Bae

**Affiliations:** ^1^Department of Neurology, Asan Medical Center, Seoul, Republic of Korea; ^2^Center for Neuroscience Imaging Research, Institute for Basic Science (IBS), Suwon, Republic of Korea; ^3^Ewha Womans University Mokdong Hospital, Ewha Womans University College of Medicine, Seoul, Republic of Korea; ^4^Dongguk University Ilsan Hospital, Dongguk University College of Medicine, Goyang, Republic of Korea; ^5^Korea University Guro Hospital, Korea University College of Medicine, Seoul, Republic of Korea; ^6^Seoul National University Bundang Hospital, Seoul National University College of Medicine, Seongnam, Republic of Korea; ^7^Department of Biostatistics, Korea University, Seoul, Republic of Korea; ^8^Artificial Intelligence Research Center, JLK Inc., Seoul, Republic of Korea; ^9^Medical Science Research Center, Dongguk University Medical Center, Goyang, Republic of Korea; ^10^Division of Stroke and Neurocritical Care, Davee Department of Neurology, Northwestern University Feinberg School of Medicine, Chicago, IL, United States; ^11^Department of Biomedical Engineering, Sungkyunkwan University, Suwon, Republic of Korea; ^12^Department of Intelligent Precision Healthcare Convergence, Sungkyunkwan University, Suwon, Republic of Korea

**Keywords:** vascular cognitive impairment, neural network, connectome, small vessel disease, amyloid deposition

## Abstract

**Background:**

Although its incidence is relatively low, delayed-onset post-stroke cognitive decline (PSCD) may offer valuable insights into the “vascular contributions to cognitive impairment and dementia,” particularly concerning the roles of vascular and neurodegenerative mechanisms. We postulated that the functional segregation observed during post-stroke compensation could be disrupted by underlying amyloid pathology or cerebral small vessel disease (cSVD), leading to delayed-onset PSCD.

**Methods:**

Using a prospective stroke registry, we identified patients who displayed normal cognitive function at baseline evaluation within a year post-stroke and received at least one subsequent assessment. Patients suspected of pre-stroke cognitive decline were excluded. Decliners [defined by a decrease of ≥3 Mini-Mental State Examination (MMSE) points annually or an absolute drop of ≥5 points between evaluations, confirmed with detailed neuropsychological tests] were compared with age- and stroke severity-matched non-decliners. Index-stroke MRI, resting-state functional MRI, and 18F-florbetaben PET were used to identify cSVD, functional network attributes, and amyloid deposits, respectively. PET data from age-, sex-, education-, and apolipoprotein E-matched stroke-free controls within a community-dwelling cohort were used to benchmark amyloid deposition.

**Results:**

Among 208 eligible patients, 11 decliners and 10 matched non-decliners were identified over an average follow-up of 5.7 years. No significant differences in cSVD markers were noted between the groups, except for white matter hyperintensities (WMHs), which were strongly linked with MMSE scores among decliners (rho = −0.85, *p* < 0.01). Only one decliner was amyloid-positive, yet subthreshold PET standardized uptake value ratios (SUVR) in amyloid-negative decliners inversely correlated with final MMSE scores (rho = −0.67, *p* = 0.04). Decliners exhibited disrupted modular structures and more intermingled canonical networks compared to non-decliners. Notably, the somato-motor network’s system segregation corresponded with the decliners’ final MMSE (rho = 0.67, *p* = 0.03) and was associated with WMH volume and amyloid SUVR.

**Conclusion:**

Disruptions in modular structures, system segregation, and inter-network communication in the brain may be the pathophysiological underpinnings of delayed-onset PSCD. WMHs and subthreshold amyloid deposition could contribute to these disruptions in functional brain networks. Given the limited number of patients and potential residual confounding, our results should be considered hypothesis-generating and need replication in larger cohorts in the future.

## Introduction

Post-stroke cognitive impairment (PSCD) can be categorized as either early or delayed in onset (Mok et al., 2017). Early-onset PSCD typically occurs 3–6 months post-stroke, affecting about 20% of patients after their first stroke (Pendlebury and Rothwell, 2009; Mok et al., 2017). In contrast, delayed-onset PSCD is characterized by cognitive decline arising 1 year or more after an initially stable post-stroke period. The prevalence of PSCD varies, ranging from 4.4 to 23.9%, depending on the observation period (Mok et al., 2017). The causal factors for these two forms of PSCD seem to diverge; early-onset PSCD is believed to stem from a complex interplay between stroke lesion characteristics and brain resilience, whereas delayed-onset PSCD is primarily attributed to cerebral small vessel disease (cSVD) and, to a lesser extent, Alzheimer’s disease (AD) pathology or recurrent stroke (Mok et al., 2017).

Although its incidence is relatively low, delayed-onset PSCD may provide valuable insights into “vascular contributions to cognitive impairment and dementia” (Snyder et al., 2015). The pattern of initial cognitive stability post-stroke, followed by later deterioration, suggests functional compensation but subsequent decompensation. cSVD, AD pathology, and stroke recurrence may underlie this decompensation process (Mok et al., 2017).

Network analysis has become instrumental in deciphering the functional architectures of the brain (Margulies et al., 2016; Bayrak et al., 2019). A strong correlation has been observed between the reduction in inter-hemispheric integration and intra-hemispheric segregation and multi-domain cognitive dysfunction in the acute stage of stroke (Siegel et al., 2016). The subsequent recovery of modularity has been linked to improvements in memory, attention, and language functions within the first year post-stroke (Siegel et al., 2018). However, a knowledge gap exists regarding delayed-onset PSCD and functional brain networks.

In this context, our study aimed to determine whether patients with delayed-onset PSCD differ from those without this condition regarding functional brain network attributes and whether such differences can be traced back to amyloid pathology or cSVD. We hypothesized that underlying amyloid pathology or cSVD might disrupt the compensation observed post-stroke, leading to delayed-onset PSCD. This disruption might manifest as a decrease in functional segregation.

## Materials and methods

This study received approval from the Seoul National University Bundang Hospital Institutional Review Board (Approval no. B-1606-352-301). Written informed consent was obtained from all eligible patients or their legally authorized representatives. All methods were performed in accordance with the relevant guidelines and regulations of the Seoul National University Bundang Hospital Ethics Committee and the Declaration of Helsinki.

### Study design and participants

**Figure 1:**
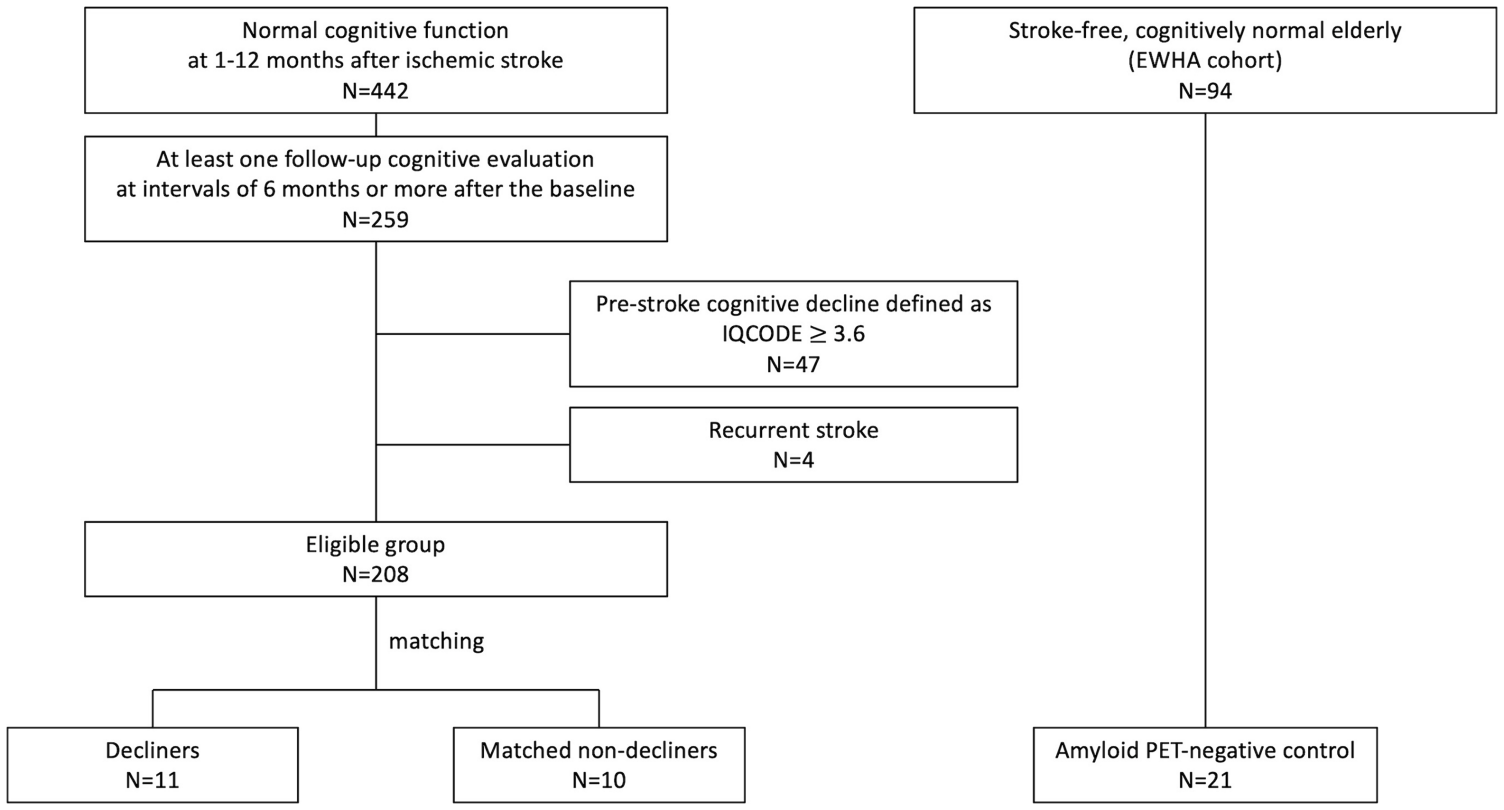
Enrollment flowchart.

We conducted a nested case–control study within a pre-established stroke cohort (Figure 1) (Kim et al., 2014) of acute ischemic stroke patients admitted to SNUBH within a week of stroke onset and registered in a prospective stroke registry from February 2007 to May 2019. The selection process began with 442 participants demonstrating normal cognition within 12 months after stroke. Cognitive function was assessed using the Mini-Mental State Examination (MMSE) and further verified through the Korean-Vascular Cognitive Impairment Harmonization Standard-Neuropsychology Protocol (K-VCIHS-NP, Supplementary material) (Kang and Na, 2003; Yu et al., 2013).

From the initial cohort, 259 patients who underwent at least one follow-up neuropsychological assessment more than 6 months after the baseline evaluation were identified. We excluded individuals suspected of pre-stroke cognitive decline [Informant Questionnaire on Cognitive Decline in the Elderly, (IQCODE) score ≥3.6, *n* = 47], even if their baseline cognitive test scores were normal, and those who experienced a recurrent stroke (*n* = 4) (Figure 1). The baseline characteristics of these 208 eligible patients are detailed in Supplementary Table S1.

Delayed-onset decliners were defined as individuals exhibiting a decline of ≥3 points annually or a drop of ≥5 points between baseline and the last MMSE scores (Hensel et al., 2007). In contrast, non-decliners were defined by a decline of one point or less per year and an absolute decline of fewer than three points (Diener et al., 2008). A prior study indicated a reliable MMSE change ranging from 2 to 4 points over 1.5 years (1.3–2.7 points per year) (Hensel et al., 2007). A significant change in the MMSE for a 5-year follow-up was reported as four points (Tombaugh, 2005). Thus, we set a criterion for a considerable change as a difference in the absolute value of ≥5 points or a decline of ≥3 points annually. Those with a significant cognitive change were re-evaluated using the K-VCIHS-NP. Non-decliners were matched with decliners by age (±3 years) and initial stroke severity [National Institutes of Health Stroke Scale (NIHSS), ±2 points].

In the 18F-florbetaben PET analysis, we selected cognitively unimpaired individuals from the community-dwelling Ewha cohort. This cohort comprised 94 cognitively unimpaired adults aged 60 or above with normal cognitive function across all cognitive domains exceeding −1 SD based on age- and education-adjusted norms (Kang and Na, 2003). Of the 62 individuals with a negative 18F-florbetaben PET scan, we selected 21 stroke-free controls by matching the decliners’ age, sex, and education. Additionally, we ensured that there were no differences in the proportion of apolipoprotein E (APOE) e4 carriers between the groups.

### Data collection

We gathered data on demographics, risk factors, and index-stroke characteristics from the registry database (Kim et al., 2014). Magnetic resonance imaging (MRI) images were downloaded from our institution’s Picture Archiving and Communication System. APOE genotyping was performed for patients who provided consent. The characteristics of stroke lesions, such as location, laterality, and multiplicity, were analyzed. Medial temporal lobe atrophy was assessed utilizing Scheltens’ visual grade (Scheltens et al., 1992). cSVD markers, including white matter hyperintensities (WMHs), lacunes, and cerebral microbleeds, were evaluated according to the Standards for Reporting Vascular changes on nEuroimaging (STRIVE) protocol using index-stroke MRI images (Wardlaw et al., 2013). All neuroimaging parameters were scrutinized by an independent rater (EBK), who has extensive neuroradiological expertise and was blinded to patient allocations. A neurology specialist (JSL) subsequently confirmed the assessments.

### WMH mapping

We produced a cumulative lesion map to provide a visual representation of WMH distributions. For the segmentation of WMHs on FLAIR sequences, we semi-automatically set regions of interest with the help of ImageQnA’s lesion growth and shrinkage algorithms (Ryu et al., 2014). To ensure consistency in comparisons across various scans, we mapped the segmented WMHs onto a standardized template—Montreal Neurological Institute 152—utilizing a mesh-warping algorithm coupled with linear interpolation. By applying a customized MATLAB code, we generated a lesion frequency map, quantifying both the voxel count and its proportion relative to total brain volume (Ryu et al., 2014).

### ^18^F-florbetaben PET

PET scans were performed exclusively on decliners, with comparative data from stroke-free controls (the Ewha cohort). The brain amyloid plaque load (BAPL) score was used to assess amyloid positivity; scores two or three denoted the presence of amyloid deposits (Barthel et al., 2011). Additionally, standardized uptake value ratio (SUVR) values were calculated for quantitative comparisons between decliners and stroke-free controls. At 90 min after the bolus intravenous administration of ^18^F-florbetaben (296 MBq, or 8 mCi), participants underwent a 20-min positron emission scan using dedicated PET/CT scanners (Biograph mCT40 or mCT64, Siemens Healthcare, Germany). A CT scan was used for attenuation correction, followed by an emission scan of the brain. PET images were reconstructed on a 400 × 400 image size with a 1 × 1 × 1.5 mm voxel size. Using 24 subsets and six iterations, images were reconstructed with ordered subset expectation maximization. A post-reconstruction Gaussian filter (full width at half maximum of 2 mm) was applied. The PET image was co-registered with the T1-MRI image. The T1-coregistered PET image was then normalized to the Montreal Neurological Institute (MNI)-152 template using the transformation matrix calculated during T1-MRI segmentation. After normalization, the SUVR was calculated using the gray matter of the cerebellum as the reference region. Using 116 gray matter regions from the automated anatomical labeling (AAL) atlas, SUVR was extracted regionally (Tzourio-Mazoyer et al., 2002). We used the averaged value of the SUVR of the four brain regions from the AAL atlas (frontal, cingulate, lateral parietal, and lateral temporal cortex) to calculate the global SUVR value. This preprocessing was accomplished with SPM12 and MATLAB 2020a (Mathworks, Natick, MA, United States). The intervals between the index stroke and ^18^F-florbetaben PET were 96.4 ± 18.0 months (mean ± standard deviation).

### Resting-state functional MRI

The protocols and preprocessing steps for functional and structural MRI can be found in the Supplementary material. We assessed functional connectivity by determining Pearson’s correlations between pairs of blood-oxygenation-level-dependent (BOLD) signals. Initially, we spatially averaged the BOLD signals of voxels within each of the 265 predefined brain regions from the Schaefer atlas (Schaefer et al., 2017).^1^ This allowed us to compute correlations between pairs of the average BOLD time series, resulting in functional connectivity matrices. We then used this region-level connectivity data to explore brain network characteristics and system segregation measures, represented graphically through spring-embedded network plots.

### Network analysis

We examined the functional network structure between decliners and non-decliners, focusing on the following: (1) conventional network attributes for global architectures, (2) system segregations of large-scale neural networks, and (3) spring-embedded graph layout for inter-network structure changes.

We evaluated the global network architecture using the Brain Connectivity Toolbox (Rubinov and Sporns, 2010). This includes measures of network integration (characteristic path length and global efficiency) and network segregation (modularity, clustering coefficient, and transitivity) (Wang et al., 2021). We calculated these measures with region-level functional connectivity matrices at various thresholds of network densities (0.05, 0.10, 0.15, 0.20). Each attribute was normalized against its null distribution, estimated by 100 iterations of random rewiring (Rubinov and Sporns, 2010). The characteristic path length was inverted, so lower values represent a worse function.

System segregation of large-scale networks quantifies a network module’s functional isolation from others. We used the equation:


$$\mathrm{SysSeg}=\frac{\bar{Z_{w}}-\bar{Z_{b}}\;}{\;\bar{Z_{w}}}\text{,}$$

where $\bar{Z_{w}}$ is the mean Fisher *z*-transformed connectivity (*r*) within the same module and $\bar{Z_{b}}$ is the mean *z*-transformed (*r*) between nodes of one module and all nodes in other modules (Chan et al., 2014). The affected large-scale networks included visual network (VN), somatomotor network (SMN), dorsal attention network (DAN), ventral attention network (VAN), limbic network (LN), frontoparietal network (FPN), and default mode network (DMN) (Yeo et al., 2011). Codes for the analyses are available on GitHub.^2^

Spring-embedded graph layout visualized qualitative characteristics in brain network composition (Gordon et al., 2017). Attractive forces were applied between connected nodes, while repulsive forces were applied to all nodes. The nodes were iteratively moved until they reached equilibrium, resulting in a layout that captures the intrinsic topology of a network. Codes for the analyses are available on GitHub.^3^

### Statistical analysis

Continuous variables were analyzed using Student’s *t*-tests or Wilcoxon rank-sum tests, while categorical variables were examined using *χ*^2^ or Fisher’s exact tests. Associations between WMH volume, ^18^F-florbetaben PET SUVR, and cognitive test scores were investigated using Pearson’s correlation. Group-level differences (i.e., decliner vs. non-decliner) in associations between WMH volume and cognitive scores were tested using linear regression with interaction terms (i.e., groups × WMH volume). We used logistic regression analysis, adjusted for age, sex, and education levels, to compare amyloid deposition patterns between decliners and stroke-free controls expressed as Group (Decliner = 1, stroke-free control = 0) = β_0_ + β_1_ age + β_2_ sex + β_3_ education + β_4_ SUVR (voxel level). In this voxel-wise PET image analysis, we used an uncorrected *p* value for exploratory purposes. All statistical analyses were performed using R version 4.0.5, with a two-sided *p*-value (< 0.05) set as the statistical threshold.

## Results

Out of 208 eligible patients, 11 (5.3%) met the criteria for delayed-onset PSCD, and there were 10 matched non-decliners. The average follow-up duration was comparable for both groups: 75.1 months for decliners and 75.4 months for non-decliners. The baseline characteristics, including features of stroke lesions and medial temporal lobe atrophy, did not differ between the two groups. The APOE e4 allele was present in one decliner and two non-decliners (Table 1).

**Table 1:** Comparisons between decliners and non-decliners.

	Decliners (*n* = 11)	Non-decliners (*n* = 10)	*P*	Stroke-free amyloid-negative controls (*n* = 21)	*P*
Baseline age, years	69.2 ± 3.63	66.7 ± 9.02	0.43	69.4 ± 4.77	0.90
Female, *n* (%)	6 (54.5)	1 (10.0)	0.06	12 (57.1)	0.89
Education, years	8.9 ± 5.8	10.9 ± 5.8	0.45	10.3 ± 3.6	0.39
Initial NIHSS (median, IQR)	3 (0, 3.5)	4.5 (2.0, 5.0)	0.11		
Baseline MMSE (median, IQR)	29 (28, 29)	27 (25, 28)	0.16	29 (28, 29)	0.55
Absolute changes in MMSE scores during follow-up (median, IQR)	−6.0 (−12.5, −6.0)	0 (−1.0, 1.0)	< 0.01		
Changes in z-scores of neuropsychological tests during follow-up					
Verbal learning test—delayed recall	−1.8 (−2.4; −1.1)	0.2 (−0.4; 0.9)	<0.01		
Boston naming test	−1.6 (−2.8; −1.3)	0.0 (−0.4; 0.6)	<0.01		
Rey complex figure test—copy	−1.8 (−4.9; −0.9)	0.0 (−0.8; 0.7)	0.02		
Semantic fluency	−1.0 (−2.4; −0.6)	0.0 (−0.5; 0.6)	0.03		
Phonemic fluency	−1.2 (−2.3; −0.8)	0.0 (−0.5; 0.4)	<0.01		
Digit symbol coding	−3.4 (−3.8; −1.4)	−0.3 (−0.9; 0.1)	<0.01		
Trail-making test—A	−0.7 (−2.3; 0.1)	0.4 (−0.8; 1.0)	0.12		
Trail-making test—B	−2.4 (−4.5; −0.8)	−0.1 (−0.9; 0.8)	0.02		
Intervals between index-stroke and last cognitive evaluations (months)	75.1 ± 24.6	75.4 ± 30.6	0.98		
Interval between baseline and last cognitive evaluations (months)	68.3 ± 26.8	67.8 ± 25.2	0.96		
APOE e4 carriers^*^	1 (9.1)	2 (20.0)	0.31	3 (14.3)	0.45
Index-stroke characteristics					
Left-sided	8 (72.7)	3 (30.0)	0.13		
Multiplicity	6 (54.5)	7 (70.0)	0.78		
Cortical involvement	6 (54.5)	3 (30.0)	0.49		
Chronic imaging variables					
Deep WMH, Fazekas grade 0–1/2/3	8 (72.7)/3 (27.3)/0 (0)	8 (80.0)/2 (20.0)/0 (0)	0.48		
Periventricular WMH, Fazekas grade 0–1/2/3	7 (63.6)/3 (27.3)/1 (9.1)	5 (50.0)/5 (50.0)/0 (0)	0.41		
WMH volume (% of brain parenchymal volume)	0.58 (0.33, 0.76)	0.52 (0.37, 0.98)	0.99		
Lacunes	2 (18.2)	4 (40.0)	0.53		
No. of lacunes, 0/1–2/3 or more	9 (81.8)/1 (9.1)/1 (9.1)	6 (60.0)/2 (20.0)/2 (20.0)			
Cerebral microbleeds	5 (45.5%)	3 (30.0%)	0.78		
No. of microbleeds, 0/1/2–4/5 or more	6 (54.5)/4 (36.4)/1 (9.1)/0 (0)	7 (70.0)/2 (20.0)/1 (10.0)/0 (0)			
Medial temporal lobe atrophy, Schelten’s grade 0–1/2/3	6 (54.6)/4 (36.4)/1 (9.1)	7 (70.0)/3 (30.0)/0 (0)	0.61		
Amyloid positivity—BAPL 2 or more	1 (9.1%)	n/a		0 (0%)	
Amyloid retention—global SUVR (mean, SD)	1.29 (0.16)	n/a			

Numbers denote mean ± standard deviations or frequencies (proportions) as appropriate. *APOE genotyping was unavailable in five participants (two decliners, two non-decliners, and one stroke-free amyloid-negative control) due to a lack of consent for genetic screening. NIHSS, National institutes of health stroke scale; MMSE, Mini-mental state examination; WMH, White matter hyperintensities; BAPL, Brain amyloid plaque load; SUVR, Standardized uptake value ratio.

The median MMSE scores at baseline were similar for both groups (29 for decliners vs. 27 for non-decliners). However, decliners reduced 6.0 points over 5.1 years, while non-decliners showed no change over 5.4 years (Supplementary Figure S1). Domain-specific z-scores on detailed neuropsychological tests consistently decreased in decliners and remained stable in non-decliners (Table 1).

### cSVD features: comparisons between decliners and non-decliners

There was no significant difference in the proportions of moderate-to-severe WMHs (Fazekas grade 2 or higher) and the volume of WMHs between decliners and non-decliners. Confluent WMHs, characterized by Fazekas grade 3, were observed in only one decliner (Table 1).

Within the decliner group, the WMH volume was significantly associated with changes in MMSE scores and final MMSE scores (rho = −0.85, *p* < 0.01; rho = −0.91, *p* < 0.01, respectively) (Supplementary Figure S2). However, no such correlation was detected within the non-decliners (rho = −0.04, *p* = 0.92; rho = −0.29, *p* = 0.41). This differential effect of WMH volume on changes in MMSE scores was confirmed in the linear regression with the interaction term (i.e., group × WMH volume). WMH volume was not significantly correlated with baseline MMSE scores (rho = −0.51, *p* = 0.11 for decliners, rho = −0.36, *p* = 0.30 for non-decliners). Mapping of WMHs from the index-stroke MRI revealed a more frequent presence of WMHs in the centrum semiovale in decliners than non-decliners (Supplementary Figure S3).

Decliners and non-decliners did not show significant differences in the presence and number of lacunes and microbleeds. Only one decliner exhibited three or more lacunes.

### Amyloid PET characteristics: comparisons between decliners and stroke-free controls

**Figure 2:**
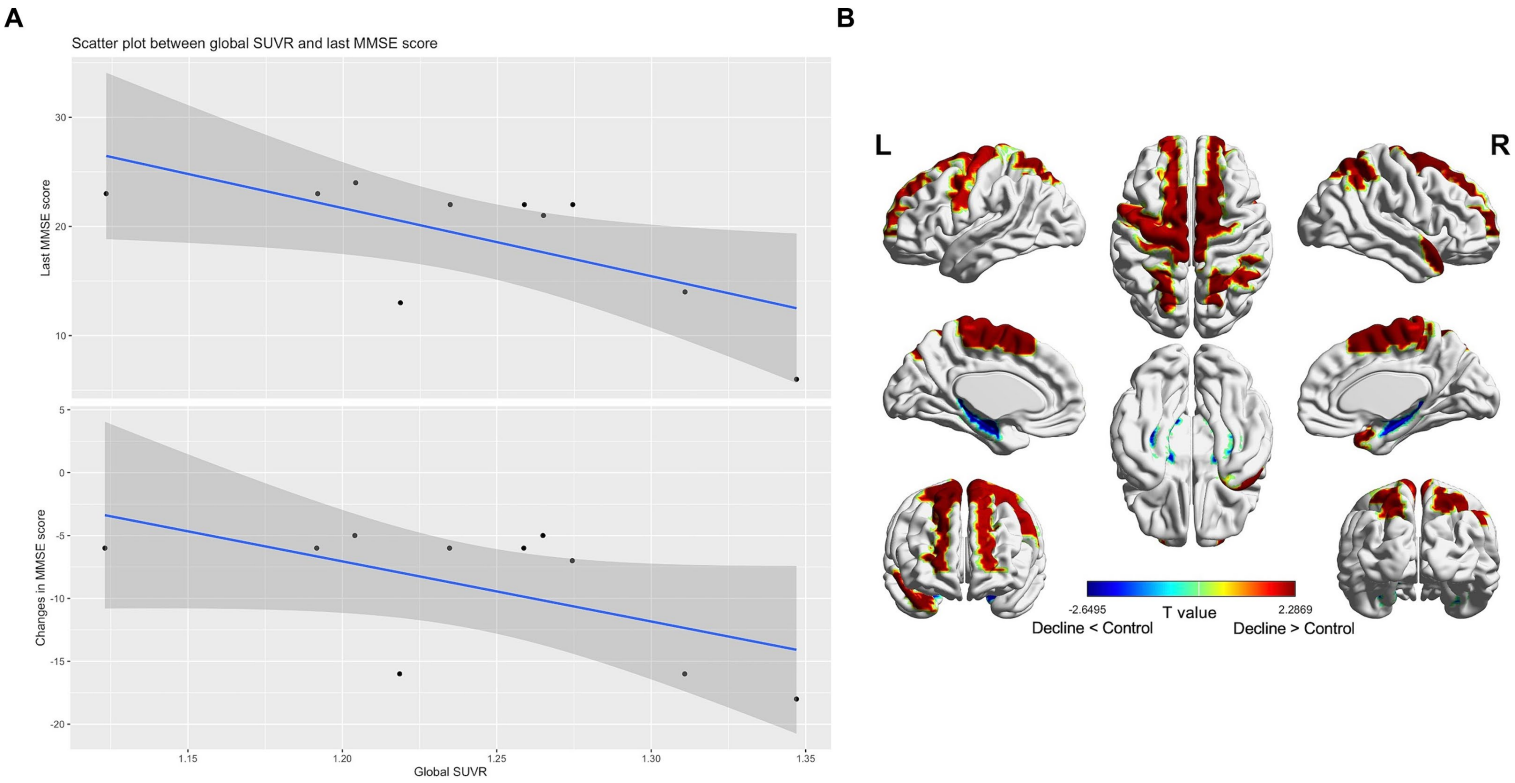
Subthreshold amyloid in patients with amyloid PET-negative delayed-onset post-stroke cognitive impairment (*n* = 10). In amyloid-negative individuals, the standard uptake value ratios were negatively correlated with the last cognitive scores and cognitive changes during follow-up **(A)**. T static map showing the difference in the topographical pattern of subthreshold amyloid deposition between the amyloid PET-negative decliners and the stroke-free, age-matched amyloid-negative controls (*n* = 21). The red color is the area where the standard uptake value ratios of the decliners increased compared to the control (thresholded by uncorrected *p* < 0.05) **(B)**.

Only one decliner exhibited amyloid PET positivity. Nonetheless, in the amyloid-negative decliners, the global SUVR value negatively correlated with both the final MMSE scores (rho = −0.67, *p* = 0.04) and changes in MMSE scores (rho = −0.58, *p* = 0.08) (Figure 2A). There was no significant correlation between the participants’ age and the SUVR value (rho = 0.09, *p* = 0.81).

In comparison to stroke-free, age-matched amyloid-negative controls (*n* = 21), the amyloid PET-negative decliners (*n* = 10) exhibited topographical patterns of subthreshold amyloid deposition in the precentral, supplementary motor, superior medial frontal, paracentral lobule, superior and inferior parietal, and superior temporal pole regions. These patterns diverged from the typical AD pattern (Figure 2B).

### Network characteristics: comparisons between decliners and non-decliners

**Figure 3:**
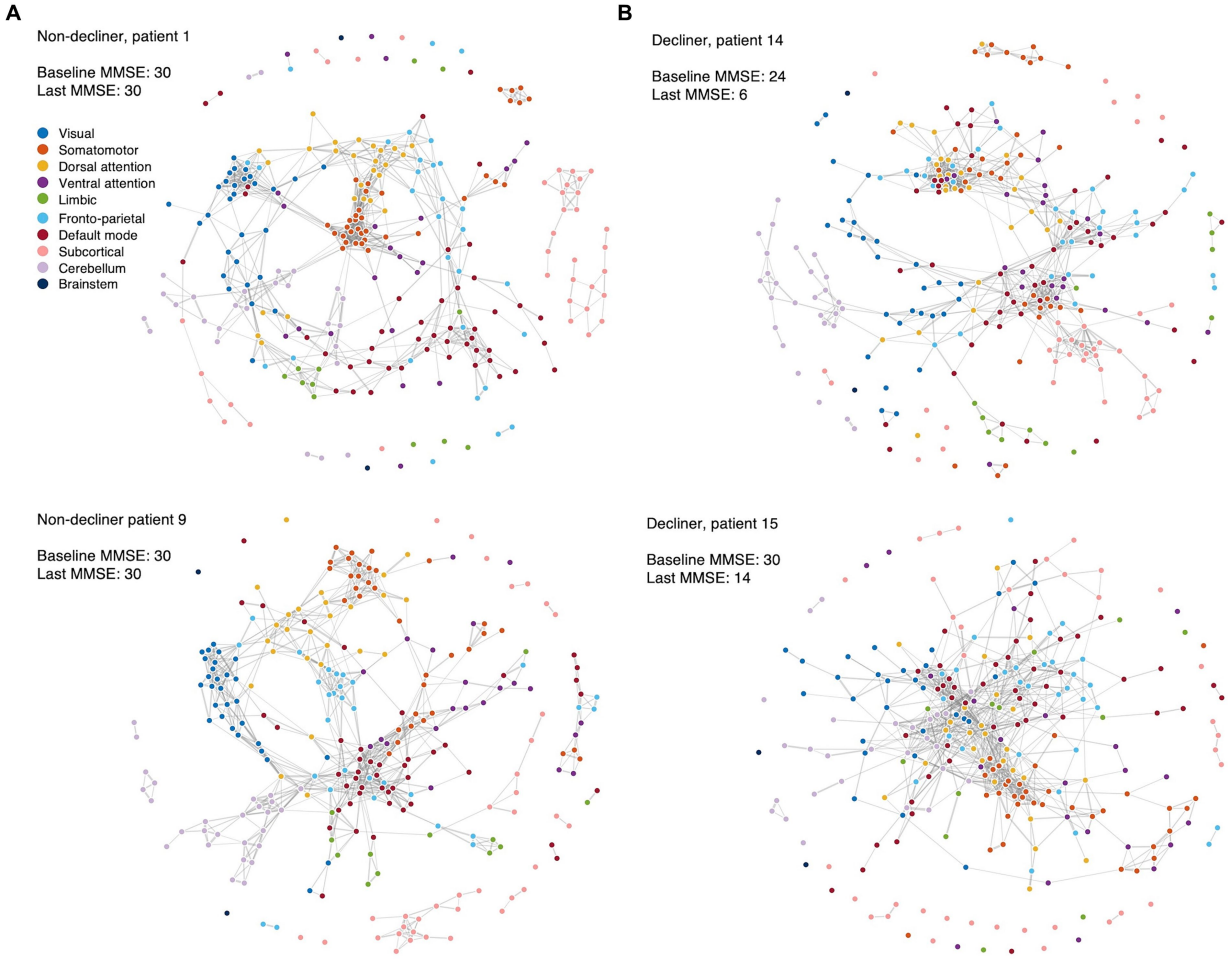
Spring-embedded graphs for the functional network compositions in the representative patients. The nodes correspond to different brain regions, with colors representing the corresponding canonical functional brain networks. The edges represent the functional connections between brain regions after thresholding. The spring-embedded graph plots present the functional brain network compositions for representative non-decliners **(A)** and decliners **(B)** at the threshold of network densities of 0.025. The plots show that the brain regions with the same canonical network were closely linked and arranged with each other **(A)**. However, representative decliners (demonstrated disrupted and intermingled canonical brain modules) **(B)**. The groups of strongly connected nodes are pulled together in the plot. Disconnected nodes are also visualized as dots without lines. Blue: visual, orange: somatomotor, yellow: dorsal attention, purple: ventral attention, green: limbic, light blue: frontoparietal, red: default mode, pink: subcortical, light purple: cerebellum, dark blue: brainstem.

When comparing the functional networks of decliners and non-decliners, we observed qualitative differences, as depicted in the spring-embedded graph (Figure 3).

In representative non-decliners (Patients 1 and 9), brain regions within the same canonical networks were closely linked and arranged (Figure 3A), similar to patterns observed in healthy participants. However, representative decliners (Patients 14 and 15) demonstrated disrupted and intermingled canonical brain modules (Figure 3B). These patterns remained broadly consistent across various thresholds of network densities and other participants (Supplementary Figures S4–6).

**Figure 4:**
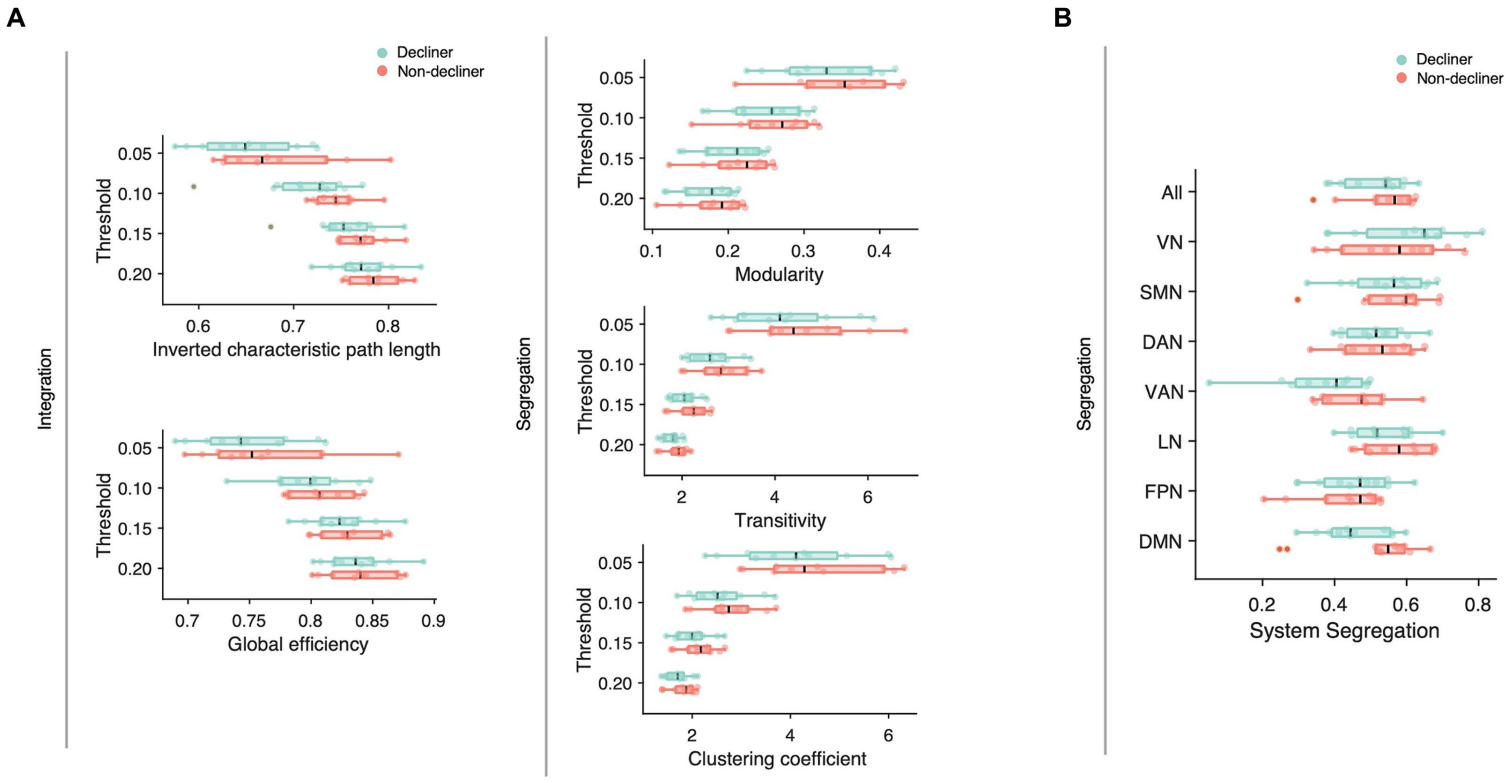
**(A)** Network attributes at multiple thresholds were compared between decliners (cyan) and non-decliners (magenta). Decliners showed lower scores in all attributes than non-decliners, but the differences were not statistically significant. Characteristic path length was inverted so that the lower value represents poorer functions to make them align with other attributes and thus help comparisons. **(B)** System segregation for different functional brain networks was compared between decliners and non-decliners. VN, visual network; SMN, somatomotor network; DAN, dorsal attention network; VAN, ventral attention network; LN, limbic network; FPN, frontoparietal network; DMN (default mode network).

We compared conventional network attributes between groups to quantitatively assess the topological differences in the functional network (Figure 4A). While no significant differences were observed between the two groups across all thresholds of network densities, all network measures were numerically lower for decliners compared to non-decliners. The effect sizes for the differences in network attributes averaged across all thresholds varied from small to large: Cohen’s *d* = 0.70, 0.32, 0.26, 0.36, 0.43 for inverted characteristic path length, global efficiency, modularity, clustering coefficient, and transitivity, respectively. Despite a lack of statistical significance due to the small sample size, we speculate that this consistent trend implies a relationship between decreased modular segregation and the resultant loss of communication efficiency with delayed PSCD, as substantiated by the spring-embedded graphs (Figure 3, Supplementary Figures S4, S5).

**Figure 5:**
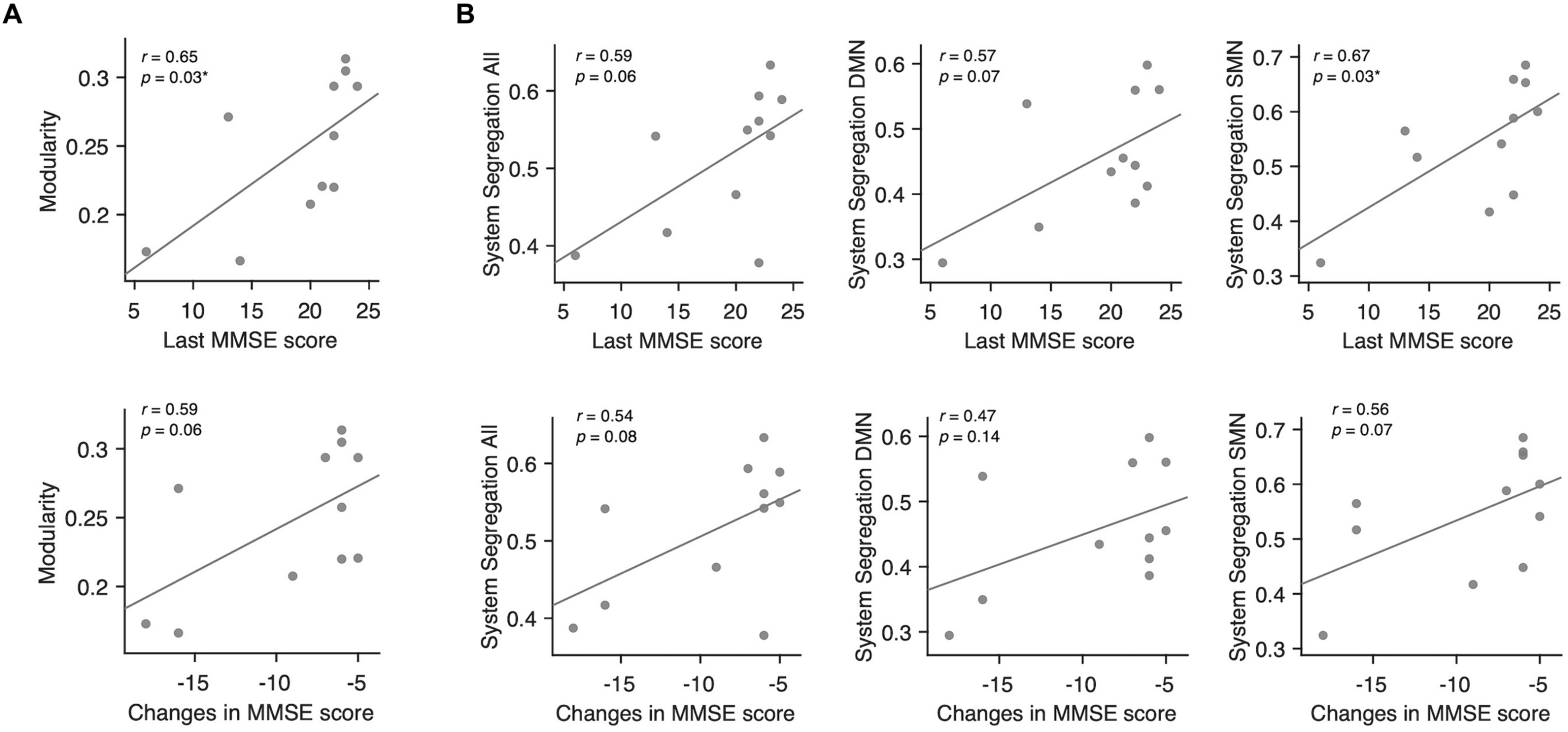
Associations between modularity, system segregation, and MMSE scores in delayed decliners. **(A)** Scatter plots for the associations between modularity averaged across all thresholds and the MMSE scores in delayed decliners, including the final MMSE score and the change in MMSE score over time. Modularity showed a significant association with the final MMSE score. **(B)** Scatter plots for the associations between system segregation across all networks, DMN, SMN, and the MMSE scores in delayed decliners, specifically the final MMSE score and the change in MMSE score over time. System segregation of the SMN showed a significant association with the final MMSE score.

Among the conventional network attributes, modularity was significantly correlated with the final MMSE scores in decliners (rho = 0.65, *p* = 0.03; Figure 5A).

To examine the network-wise contribution of modular segregation to the delayed-onset PSCD, we calculated system segregation, which quantifies the relative difference between within-network and between-network connectivity. Despite no significant difference in system segregation between decliners and non-decliners (Figure 4B), the final MMSE scores in decliners displayed a strong but marginally significant association with system segregation for all canonical networks and DMN (rho = 0.59, *p* = 0.06; rho = 0.57, *p* = 0.07) and a significant association with system segregation for SMN (rho = 0.67, *p* = 0.03) (Figure 5B). This suggests the role of SMN functional specialization in delayed PSCD. The relationships between network attributes and other cognitive scores, except MMSE, were not significant (Supplementary Figure S7).

### Associations among network attributes, WMH volume, and amyloid PET SUVR

We calculated correlations between network attributes, WMH volume, and subthreshold PET SUVR in amyloid-negative decliners (*n* = 10) and observed a strong negative correlation between both WMH volume and PET SUVR with SMN system segregation (rho = −0.76, *p* < 0.01; rho = −0.78, *p* < 0.01; respectively) (Supplementary Table S2). The WMH volume showed marginal associations with the system segregation of all large-scale networks (rho = −0.60, *p* = 0.07) and FPN (rho = −0.61, *p* = 0.06). Similarly, amyloid PET SUVR showed marginal associations with system segregation across all canonical networks (rho = −0.62, *p* = 0.06), DAN (rho = −0.62, *p* = 0.06), VAN (rho = −0.57, *p* = 0.09), and FPN (rho = −0.57, *p* = 0.08).

## Discussion

We found that approximately 5% of stroke survivors who initially exhibited normal cognitive function developed delayed cognitive impairment around 6 years post-stroke. This cognitive decline significantly correlates with network characteristics such as modularity and system segregation. Furthermore, a significant association was observed between WMH volume and cognitive changes among decliners. Notably, among the amyloid-negative decliners, a substantial inverse correlation was found between the global SUVR of ^18^F-Florbetaben PET and final MMSE scores. These results suggest that our hypothesized interrelationship between cSVD, amyloid pathology, and functional segregation as a compensatory mechanism after stroke may influence delayed PSCD.

Our findings indicate that underlying WMHs and subthreshold amyloid pathology might jointly contribute to delayed-onset PSCD. If either amyloid pathology or WMHs operate independently, symptoms only appear when the severity of either pathology surpasses a particular threshold. In our study, confluent WMHs were seen in only one decliner, and WMH volume was not exceedingly high (media*n* = 0.58 in decliners and 0.52 in non-decliners, as a percentage of brain parenchymal volume) (Ryu et al., 2016). Likewise, while only one decliner displayed amyloid PET positivity, there was a substantial correlation between the SUVR value and cognitive scores, even at subthreshold levels. Previous research has suggested that subthreshold amyloid pathology potentially impacts delayed cognitive decline if it interacts with vascular pathology (Kapasi and Schneider, 2016). Patients with delayed-onset PSCD in our study exhibited significant deposition in the precentral gyrus, supplementary motor area, superior medial frontal cortex, paracentral lobule, superior and inferior parietal lobules, and superior temporal pole. This contrasts with the initial sites of amyloid accumulation in typical AD patients: the medial frontal, medial parietal, and lateral temporal–parietal lobes (Grothe et al., 2017). It has been reported that amyloid deposition in cases of cerebral amyloid angiopathy (CAA) is relatively increased in the occipital region (Charidimou et al., 2018). Considering this, it seems unlikely that the delayed-onset PSCD patients in our study are concurrently exhibiting typical early-stage AD or CAA. Nonetheless, due to the limited number of patients, the statistical interpretation of regional amyloid deposition patterns should be approached cautiously. Considering these points, along with the conflicting results reported in previous studies regarding the impact of amyloid pathology on the delayed onset of PSCD (Wollenweber et al., 2016; Liu et al., 2018) it may result from a complex interaction of multiple factors rather than a single independent influence.

We hypothesize that WMHs and amyloid pathology may contribute to delayed cognitive decline by altering the intracerebral network environment. Previous studies have indicated that WMH-induced white matter tract damage and amyloid pathology can result in neural network dysfunction, which, in turn, can negatively impact cognitive function (Taylor et al., 2017; Crockett et al., 2021; Coenen et al., 2023). Recent studies have also shown that amyloid pathology compromises white matter tract integrity (Collij et al., 2021). Our results are consistent with these findings, showing that the WMH volume and subthreshold PET SUVR are associated with decreased SMN system segregation. Although the decliners did not show statistically significant differences in conventional network attributes compared to the non-decliners, likely due to the small sample size, there were consistent trends indicating that these network attributes were less favorable in the decliners in both group comparisons and correlation analyses.

Previous studies suggested a pathophysiological link between neural network segregation, WMHs, and amyloid pathology, pointing to these factors as potential culprits for cognitive decline (Mok et al., 2016, 2017). For example, disruptions of functional segregation following a stroke could lead to global cognitive dysfunction (Siegel et al., 2018). Patients with higher WMH scores exhibited increased shortest path lengths, decreased clustering coefficient values, and reduced node efficiency, which significantly correlated with lower total Montreal Cognitive Assessment (MoCA) scores (Wang et al., 2022). In line with this, our results showed that modularity was negatively associated with cognitive outcomes and lower in the decliners than non-decliners. This finding is further supported by the low clustering coefficient and transitivity observed in the decliners and the decreased system segregation across all canonical networks (Figure 4). Our results also suggest that the functional structures of each canonical network may intermingle with each other, as seen in the spring-embedded graphs (Figure 3) (Gordon et al., 2017).

When we examined specific canonical networks, the system segregation of the SMN was significantly associated with cognitive outcomes in the decliners (Figure 5B). The SMN is related to somatosensory and action functions and is thus crucial for interacting with the surrounding environment. In old age, the relative contribution of SMN to the dynamic functional state of the resting brain decreases (Zhang et al., 2021), and a decline in the within-network connectivity of the SMN has been associated with poor cognitive performances (Geerligs et al., 2014). Previous studies have also suggested that disruption of sensorimotor networks due to WMH may contribute to overall cognitive deficits in older adults with cerebral small vessel disease (Crockett et al., 2021). Furthermore, the connectivity of the SMN in stroke patients significantly contributes to multitasking learning (Siegel et al., 2016).

Using a prospective stroke registry database, we identified 208 patients who had experienced a mild stroke but initially demonstrated normal cognitive function (Ryu et al., 2016). Over an average follow-up of 75 months, 5.3% (11 of 208) exhibited delayed onset PSCD. This rate is consistent with a previous study by Mok et al. (2016) wherein approximately 4.4% of 919 patients displayed delayed-onset dementia over a 2.6-year follow-up period (Mok et al., 2016). We found commonalities in our cohort’s baseline characteristics, and Mok et al.’s: mean age was 69.1 ± 10.6 vs. 68.6 ± 11.4; and female participants were 39.9 vs. 42.8% (Mok et al., 2016). However, neuroimaging characteristics of delayed decliners differed. Mok et al. (2016) found that confluent WMHs and three or more lacunes predicted post-stroke delayed-onset dementia. In contrast, only one decliner had these traits in our study. These discrepancies are likely attributed to the unique profiles of decliners in each study; our decliners were younger (69.2 vs. 76.2 years), had more years of education (8.9 vs. 4.1), and scored higher on the baseline MMSE (29 vs. 20) compared to Mok et al. (2016) decliners. These observations suggest that factors influencing delayed-onset PSCD may differ depending on the population of interest.

Our study has limitations. Despite the substantial number of stroke patients followed for over 6 years, the sample size was limited due to the low rate of cognitive decline in a single-center study. Furthermore, many variables, such as lesion location and size, were not closely matched between the groups, potentially leading to residual confounding. As a result, this study should be viewed as hypothesis-generating, with a need to validate the findings in larger-scale studies. Additionally, due to research funding limitations, we could not collect ^18^F-Florbetaben PET data for the non-decliners in the study. Despite these limitations, we aimed to shed light on the characteristics and mechanisms underlying the worsening of cognition in patients with delayed-onset PSCD. This aim was achieved by including matched non-decliners and stroke-free elderly controls and obtaining functional MRI data.

In conclusion, after an almost 6-year observation period, the volume of WMHs, the presence and degree of subthreshold amyloid deposition, and alternations in functional brain networks emerged as possible factors in understanding delayed cognitive decline following stroke. Our findings suggest that delayed-onset PSCD may result from a complex interaction of multiple factors rather than a single cause. Understanding the stroke connectome and dynamic changes in brain networks during the acute and chronic phases of stroke holds promise for predicting cognitive changes during recovery and understanding how preventive therapy may be individually tailored.

## Data Availability

The raw data supporting the conclusions of this article will be made available by the authors, without undue reservation.

## Footnotes

^1^ https://github.com/cocoanlab/cocoanCORE/blob/master/Canonical_brains/Schaefer/Schaefer_265_combined_2mm.nii

^2^ https://github.com/cocoanlab/cocoanCORE/blob/master/Network_analysis/system_segregation.m

^3^https://github.com/cocoanlab/cocoanCORE/blob/master/Visualization/vis_network.m
