# Supplementary material for: Subthreshold amyloid deposition, cerebral small vessel disease, and functional brain network disruption in delayed cognitive decline after stroke
Source: Front Aging Neurosci. 2024 Sep 16;16:1430408. doi: 10.3389/fnagi.2024.1430408 (PMC11439663; doi:10.3389/fnagi.2024.1430408)
Supplement: Supplementary file 1 [file Data_Sheet_1.docx]

**Supplemental Materials**

Supplemental Methods

Figure S1. Spaghetti plot for the cognitive changes of individual participants

Figure S2. Scatter plot between white matter hyperintensity volumes and cognitive scores

Figure S3. Cumulative lesion mapping for white matter hyperintensities between decliners and non-decliners

Figure S4. Spring-embedded figures for the representative non-decliners with various network densities

Figure S5. Spring-embedded figures for the representative decliners with various network densities

Figure S6. Spring-embedded figures for all participants at the threshold of network densities of 0.025

Figure S7. Correlation matrix between network attributes and cognitive scores

Table S1. Baseline characteristics of eligible patients

Table S2. Correlations between WMH volumes, 18F-Florbetaben PET SUVR and network attributes in amyloid-negative delayed decliners

Supplemental References

**Supplemental Methods**

**Detailed Neuropsychological Assessment**

The K-VCIHS-NP was a Korean version of the 60-min neuropsychology protocol of the VCIHS proposed by the National Institute of Neurological Disorders and Stroke and the Canadian Stroke Network.([Hachinski et al., 2006](" \l "biblioRef00); [Yu et al., 2013](#biblioRef02)) It comprises four cognitive domains: frontal executive function, language, visuospatial function, and memory. Detailed tests for each cognitive domain were 1) Controlled Oral Word Association Test, Digit Symbol Coding, Trail Making Test-Elderly’s version for the frontal executive domain; 2) Short Form of the Boston Naming Test for language domain; 3) Rey Complex Figure Test: Copy for the visuospatial domain; and 4) Seoul Verbal Learning Test for memory domain. All the cognitive tests were validated and standardized in Korean. Trained clinical neuropsychologists administered a series of tests. A score on each cognitive test was transformed into a standardized Z score.

**Stroke-free control group: Ewha cohort**

Detailed exclusion criteria for the Ewha cohort were as follows: 1) suspected or confirmed mild cognitive impairment or dementia; 2) suspected or confirmed major neurological disorders such as stroke or psychiatric illnesses, including major depressive disorders; 3) contraindications to magnetic resonance imaging (MRI); 4) visual or hearing impairments severe enough to interfere with questionnaire response; 5) history of medications that could affect cognitive and emotional functions in the last three months, and or 6) other significant medical problems. Of the 94 participants, 76 underwent 18F-florbetaben PET scans, and 62 (81.6%) exhibited negative amyloid deposition (brain amyloid plaque load [BAPL] 1).

**Magnetic Resonance Imaging for index stroke**

A 3.0-Tesla magnetic resonance imaging scanner (Achieva, Philips Healthcare, Eindhoven, The Netherlands) was used for this study. The MRI protocols consisted of diffusion-weighted imaging (DWI), axial T1- and T2-weighted spin-echo, fluid-attenuated inversion recovery imaging (FLAIR), gradient-echo imaging, and coronal T1-weighted spin-echo imaging. FLAIR imaging was obtained using a fast-spin echo sequence. Acquisition parameters for FLAIR images were set as follows: repetition time, 11,000 ms; echo time, 125 ms; inversion time, 2,800 ms; slice thickness, 5 mm; intersection gap, 1 mm; matrix, 512 x 512; flip angle, 90 degrees. DWI imaging was obtained using an EPI-spin echo sequence. Acquisition parameters for DWI images were set as follows: repetition time, 5,000 ms; echo time, 50 ms; diffusion b-value 1,000; slice thickness, 5 mm; intersection gap, 1 mm; matrix, 256 x 256; flip angle, 90 degrees.

**Magnetic Resonance Imaging research protocol**

Functional and structural MRI data were collected using a Philips 3T Achieva MRI scanner. The rs-fMRI data were acquired with the following parameters: TR = 3,000 ms, TE = 30.0 ms, field of view = 224 mm, 128$\times$128 matrix, 1.75$\times$1.75$\times$3.00 mm3 voxels, 45 interleaved slices, number of volumes = 180. During the rs-fMRI acquisition, patients were instructed to keep their eyes open. High-resolution T1-weighted structural images were obtained using specific paraments including field of view = 240 mm, 480$\times$480 matrix, 1.00$\times$0.50$\times$0.50 mm3 voxels, 175 slices.

**Assessment of Functional Brain Network**

**Preprocessing of Resting-State Functional MRI Data**

The rs-fMRI images underwent preprocessing using SPM12. This encompassed a series of steps such as slice-timing correction, realignment, co-registration, normalization, and smoothing. For image preprocessing, we visually confirmed the acute stroke lesions using diffusion-weighted images obtained at admission and delineated the lesion mask on the volumetric T1 images using MRIcron. These lesion masks were used for the subsequent image preprocessing. Any image distortions and motion artifacts were visually reassessed. For rs-fMRI, we processed the data using a pipeline based on SPM12. First, all DICOM files were converted to NIfTI format for further processing. For quality assessment of the functional images, implicit masks were created, and the mean images were saved. Outliers were identified based on the mean signal intensity, Mahalanobis distances, and the root mean square of successive differences across volumes. Images exceeding 3 standard deviations from the global mean were considered outliers. Images that exceeded 10 mean absolute deviations (MADs) based on moving averages with full width at half maximum (FWHM) 20 image kernels were also identified as outliers for signal intensity and Mahalanobis distances. Each time point identified as an outlier by the outlier detection method was included as a nuisance covariate. Functional images were slice-timing corrected and motion-corrected (realigned). Volumetric T1 images were then coregistered to the functional image and normalized to the MNI template (2*2*2 mm^3^) using a unified segmentation-normalization algorithm with masked lesions.18 These warping parameters were then applied to the functional images, smoothed with the 5-mm FWHM smoothing kernel. Nuisance covariates were regressed out, including outlier time-points, 24 head motion parameters (x, y, z, roll, pitch, and yaw, their mean-centered squares, their derivatives, and squared derivative), linear drift, and principal components of white matter and ventricle signals (with five principal components each). Spatiotemporal outlier voxels (intensity value < median – 5 SD or > median + 5 SD) were further Winsorized, and a band-pass filter (0.008-0.1 Hz) was applied. Codes that were used for preprocessing can be found at <https://github.com/cocoanlab/humanfmri_preproc_bids/>. For subsequent image analyses, the presence or absence of errors in images, including head motions and registration accuracy, were evaluated for all study participants.

**Network thresholding**

To visualize brain networks using a spring-embedded graph layout, we applied proportional thresholding of the region-level functional connectivity matrices at varying network thresholds (0.025, 0.05, 0.075, 0.1). We used proportional thresholding at network thresholds 0.05, 0.1, 0.15, and 0.2 to calculate conventional network attributes.

**Figure S1. Spaghetti plot for the cognitive changes of individual participants**


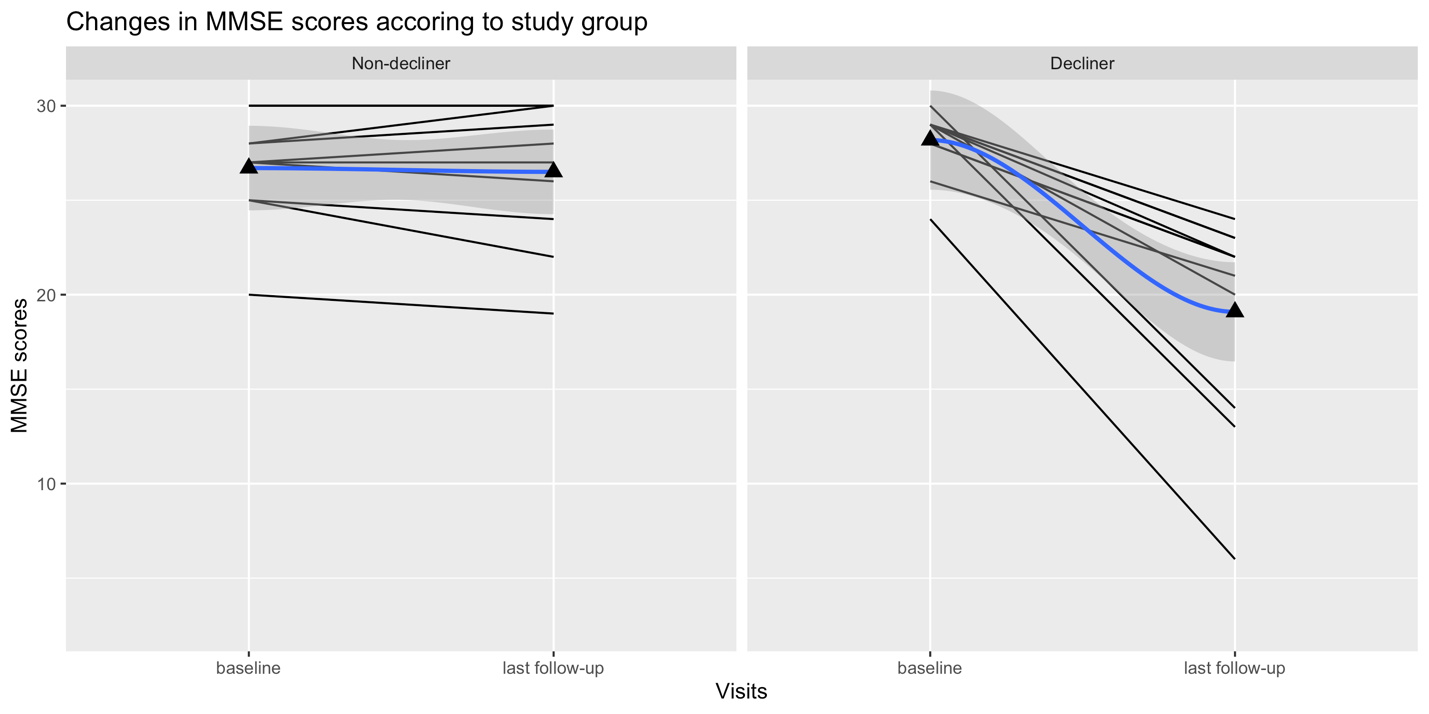


**Figure S2. Scatter plot between white matter hyperintensity volumes and cognitive scores in decliners**

**
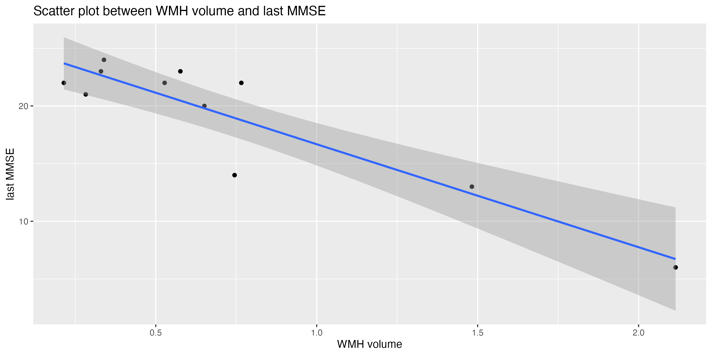
**

**
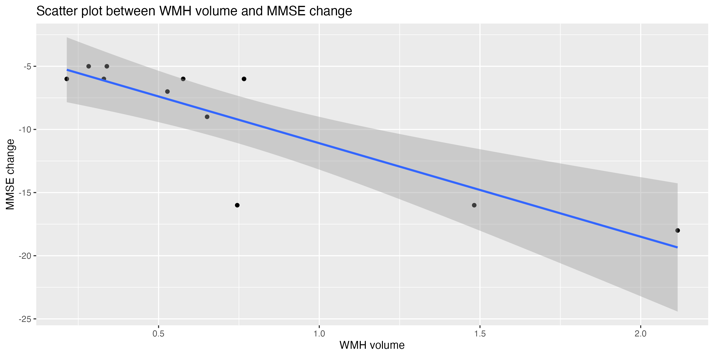
**

**Figure S3. Cumulative lesion mapping for white matter hyperintensities between decliners and non-decliners**

**
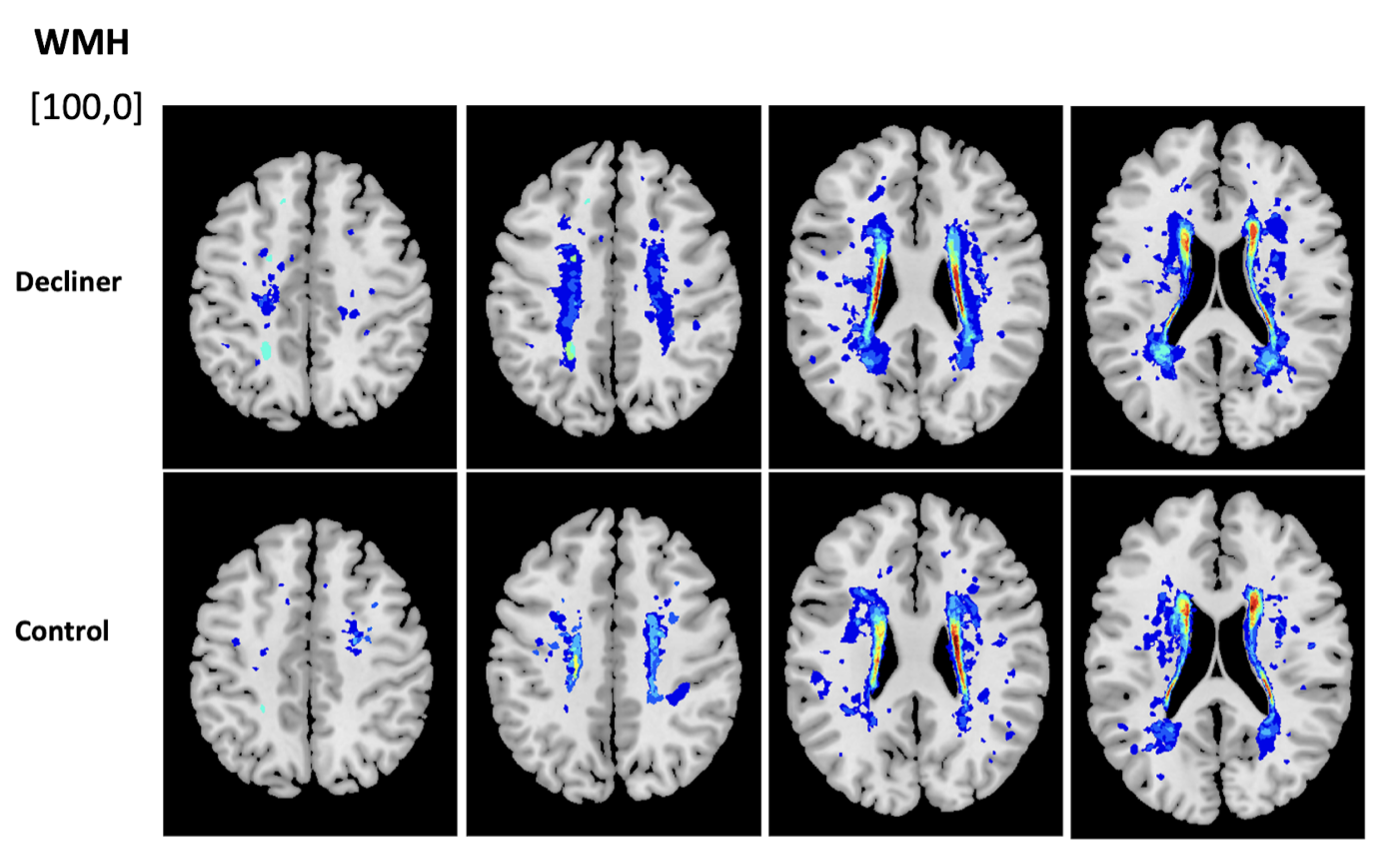
**

**Figure S4. Spring-embedded figures for the representative non-decliners with various network densities**

**
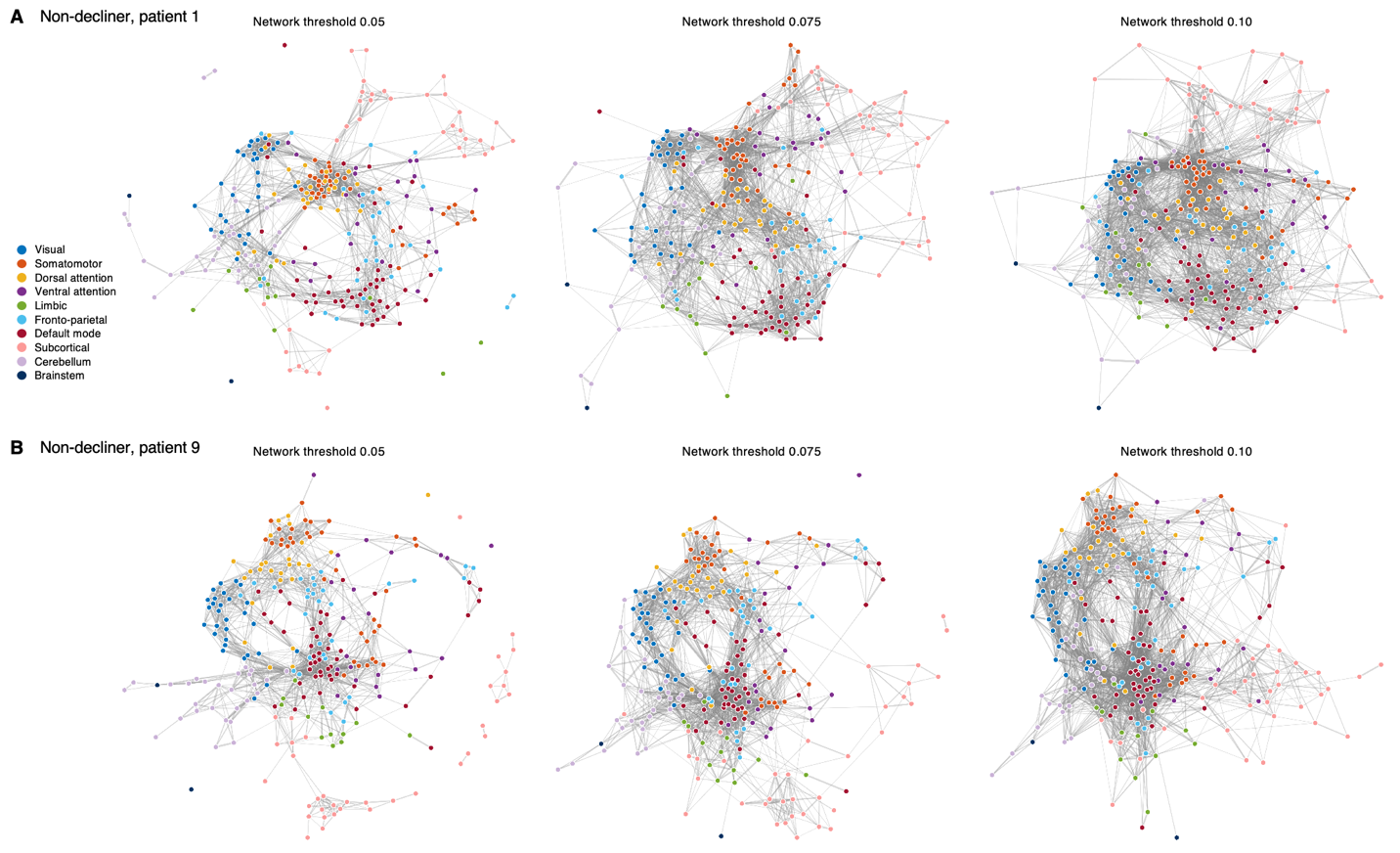
**

**Figure S5. Spring-embedded figures for the representative decliners with various network densities**

**
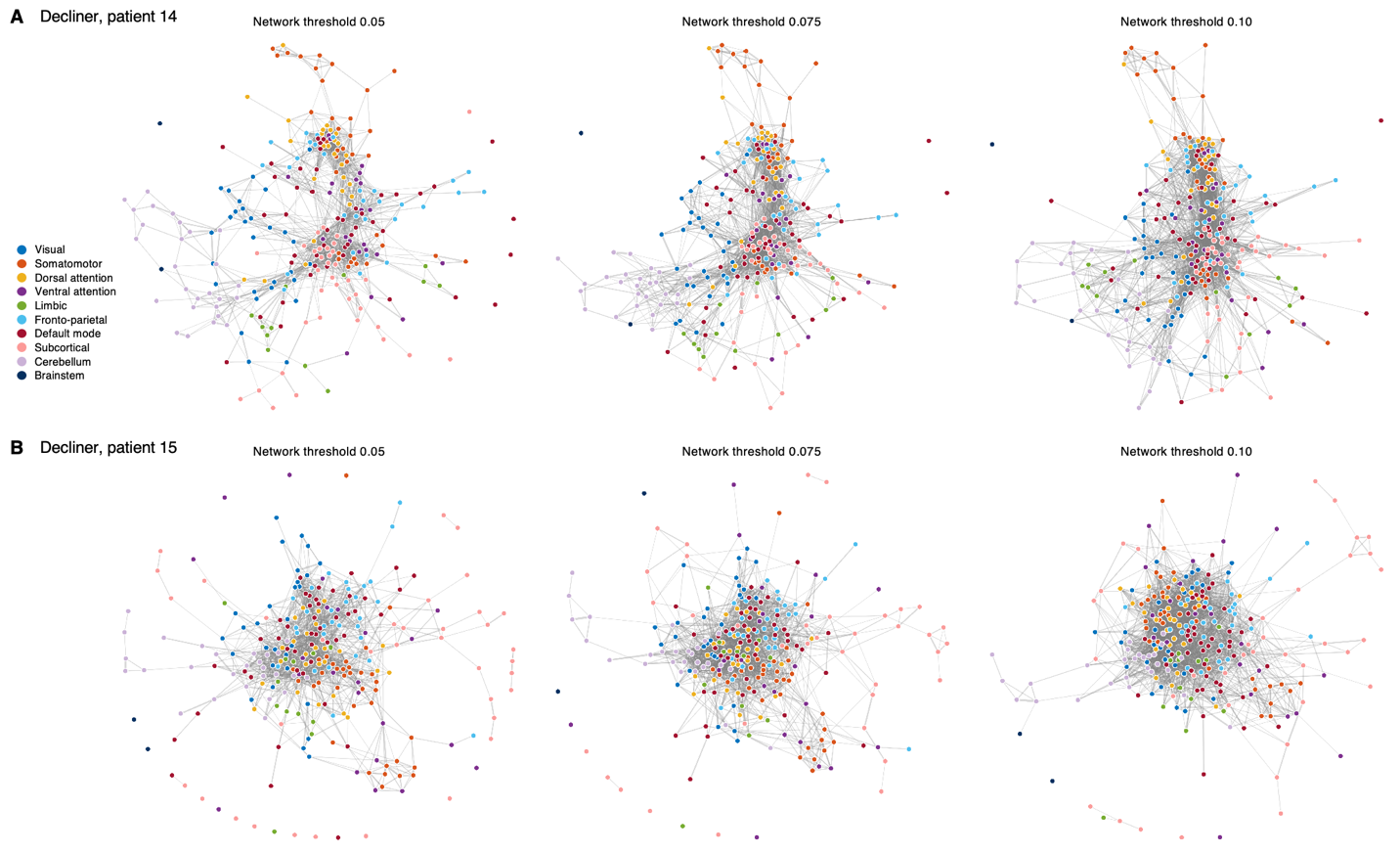
**

**Figure S6. Spring-embedded figures for all participants at the threshold of network densities of 0.025**

**
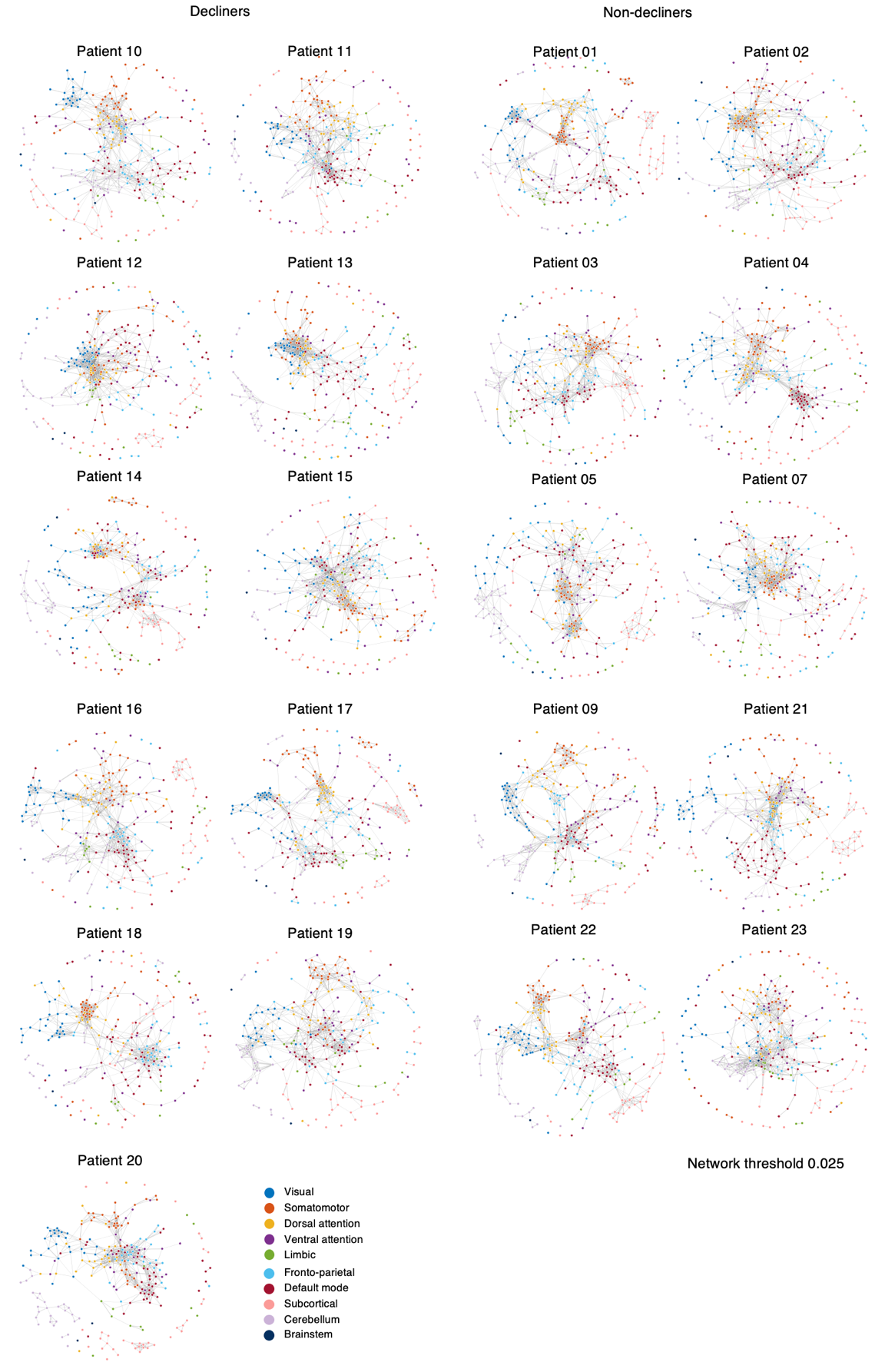
**

**Figure S7. Correlation matrix between network attributes and cognitive scores**

~~
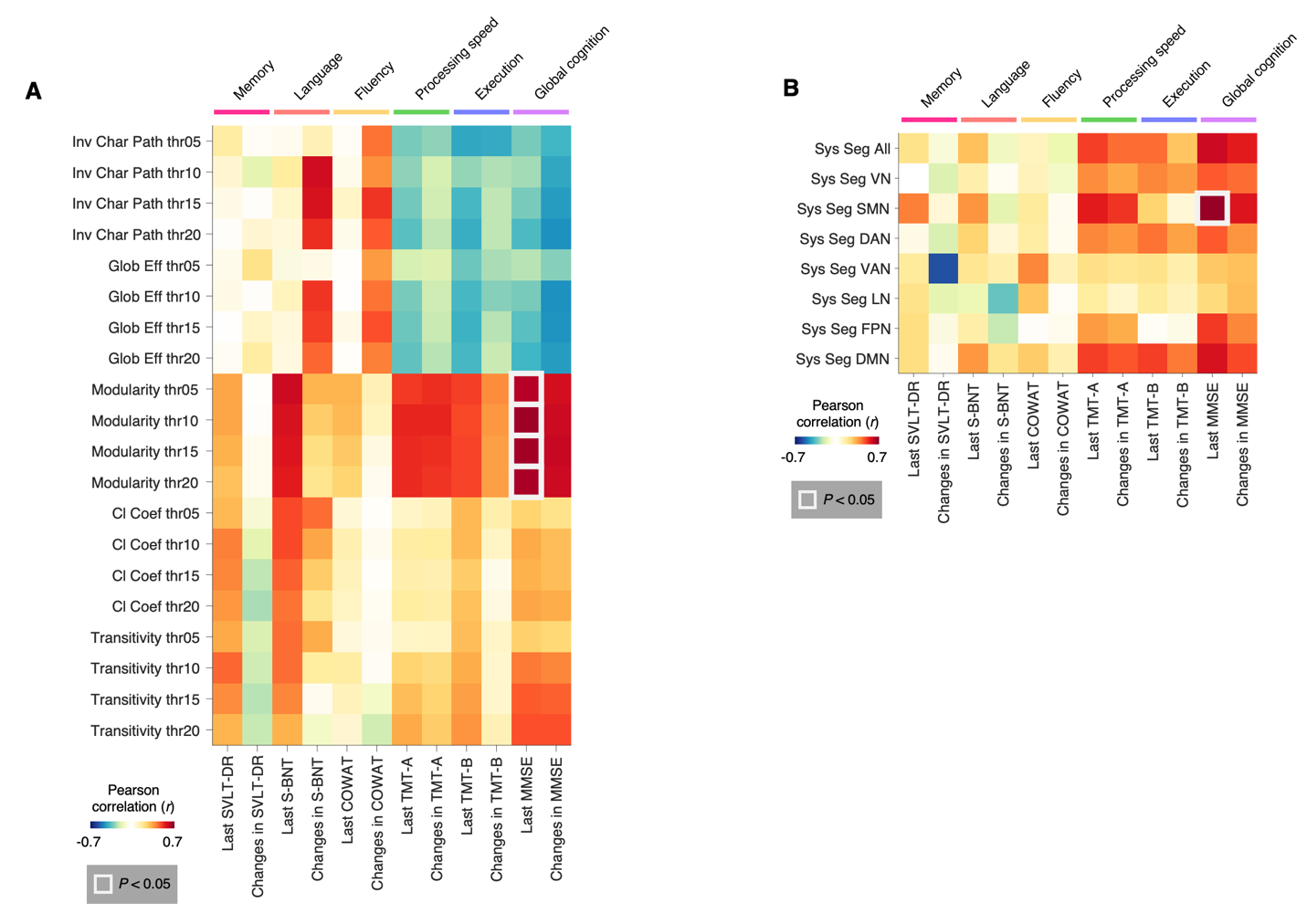
~~**(A)**. Correlation matrix between network attributes at various threshold and cognitive scores. All cognitive variables have been transformed to represent a good function with high values. Statistically significant results (*p* < 0.05) were marked in white squares. **(B)**. Correlation matrix between system segregations of canonical networks and cognitive scores.

Abbreviations: Inv Char Path (inverted characteristic path length), thr (threshold), Glob Eff (global efficiency), Cl Coef (clustering coefficient), SVLT-DR (Seoul verbal learning test-delayed recall), S-BNT (short version of Boston naming test), COWAT (controlled oral word association test), TMT (trail-making test), MMSE (mini-mental state examination), Sys Seg (system segregation), VN (visual network), SMN (somatomotor network), DAN (dorsal attention network), VAN (ventral attention network), LN (limbic network), FPN (frontoparietal network), DMN (default mode network)

**Table S1. Baseline characteristics of eligible patients**

|  | Eligible group  (n=208) |
| --- | --- |
| Baseline age, years | 69.1 $\pm$ 10.6 |
| Female, n (%) | 83 (39.9) |
| Education, years | 9.9 $\pm$ 5.6 |
| Baseline MMSE (median, IQR) | 28 (27, 29) |
| Absolute changes in MMSE scores during follow-up (median, IQR) | 0 (-2.0, 1.0) |

Numbers denote mean ± standard deviations or median (interquartile ranges) for continuous variables or frequencies (proportions) for categorical variables.

Abbreviations: MMSE (Mini-Mental State Examination), IQR (interquartile range)

**Table S2. Correlations between WMH volumes, ^18^F-Florbetaben PET SUVR, and network attributes in amyloid-negative delayed decliners**

|  | **WMH volumes** | | **Amyloid PET SUVR** | | |
| --- | --- | --- | --- | --- | --- |
| **Variables** | **rho^*^** | ***P***^†^ | **rho^*^** | ***P***^†^ |  |
| Network attributes |  |  |  |  |  |
| Characteristic path length | -0.46 | 0.22 | -0.22 | 0.57 |  |
| Global efficiency | 0.42 | 0.26 | 0.35 | 0.36 |  |
| Clustering coefficient | -0.16 | 0.68 | -0.62 | 0.07 |  |
| Modularity | -0.48 | 0.19 | -0.83 | <0.01 |  |
| Transitivity | -0.30 | 0.43 | -0.65 | 0.06 |  |
| System segregation |  |  |  |  |  |
| Canonical-all | -0.60 | 0.09 | -0.61 | 0.08 |  |
| Visual network | -0.40 | 0.28 | -0.46 | 0.22 |  |
| Somatomotor network | -0.77 | 0.02 | -0.78 | 0.01 |  |
| Dorsal attention network | -0.29 | 0.44 | -0.62 | 0.08 |  |
| Ventral attention network | -0.16 | 0.68 | -0.67 | 0.0502 |  |
| Limbic network | -0.23 | 0.55 | -0.16 | 0.68 |  |
| Frontoparietal network | -0.72 | 0.03 | -0.64 | 0.06 |  |
| Default mode network | -0.46 | 0.21 | -0.51 | 0.16 |  |

^*^ Partial correlation coefficients after controlling for age

^†^ Pearson’s correlation tests

**Supplemental References**

Hachinski, V., Iadecola, C., Petersen, R. C., Breteler, M. M., Nyenhuis, D. L., Black, S. E., et al. (2006). National Institute of Neurological Disorders and Stroke–Canadian Stroke Network Vascular Cognitive Impairment Harmonization Standards. *Stroke* 37, 2220–2241. doi: 10.1161/01.str.0000237236.88823.47

Tzourio-Mazoyer, N., Landeau, B., Papathanassiou, D., Crivello, F., Etard, O., Delcroix, N., et al. (2002). Automated Anatomical Labeling of Activations in SPM Using a Macroscopic Anatomical Parcellation of the MNI MRI Single-Subject Brain. *Neuroimage* 15, 273–289. doi: 10.1006/nimg.2001.0978

Yu, K.-H., Cho, S.-J., Oh, M. S., Jung, S., Lee, J.-H., Shin, J.-H., et al. (2013). Cognitive Impairment Evaluated With Vascular Cognitive Impairment Harmonization Standards in a Multicenter Prospective Stroke Cohort in Korea. *Stroke* 44, 786–788. doi: 10.1161/strokeaha.112.668343
